# Charles Davies Sherborn and the “Indexer’s Club”

**DOI:** 10.3897/zookeys.550.9697

**Published:** 2016-01-07

**Authors:** Neal L. Evenhuis

**Affiliations:** 1 Bishop Museum, 1525 Bernice Street, Honolulu, Hawaii 96817, USA

**Keywords:** Indexing, bioinformatics, zoology, biography

## Abstract

The first few words of the title of this symposium are “Anchoring Biodiversity Information”. In order to properly anchor anything for a long-lasting future, a solid foundation needs to have been laid. For the zoological portion of biodiversity information, that firm foundation is best exemplified in the works of Charles Davies Sherborn. This man, like others of his ilk, was intimately focused on indexing names. This incredible focus was a life-long passion for him and culminated in his 9500-page *Index Animalium* of over 400,000 names of animals. This *Index* represents not only one of the most prodigious efforts in publication by a single man and the single most important reference to names in zoology, but a permanent legacy to the efforts of an indexer that proved to be an inspiration to many.

“*Nomina si pereunt, perit & cognitio rerum*.” [If the names are lost, the knowledge also disappears.]

– C. Linnaeus, 1775, *Bigae Insectorum* , p. 305

## Introduction

Before we go into the life and work of Sherborn, a bit of an introduction needs to be made as to just who indexers are, and what makes them index. I call this group of individuals the “*Indexer’s Club*”. It is a unique gathering of like minds that for some reason have found comfort in essentially making order out of disorder for large groups of things. There are professional indexing societies in Australia and New Zealand, Britain, Canada, China, Germany, the Netherlands, and Southern Africa and an international quarterly journal, The Indexer, which covers a wide range of indexing-related matter. Technically, “indexes” and “lists” are two different things, but for convenience in this paper, I am lumping the two into “indexing” *sensu lato.*

In finding a way to make order out of an otherwise chaotic array of things, indexing is not necessarily making a long list of names in alphabetical order. It can be as simple as making a shopping list, a list of chores, a list of synonyms by category, a list of phone numbers, a list of birth-stones by month, or maybe even a list of past lovers (in chronological order, of course) or it can be a very complex and onerous task involving large numbers of unsorted items. We are all indexers in that we have made some of the lists just mentioned. Some lists may have been out of simple curiosity (“based on the data from the exams of my students that I have graded, I wonder who is at the top of the list in my class”); others may have been made because it helped us in some way (“based on the data from the exams of my students I have graded, I wonder if I may get a promotion”). Whether the user is us or others, simply said, indexers facilitate the various users of data to expedite their work by forming an ordered methodology to find what is being sought.

**Figure 1.**
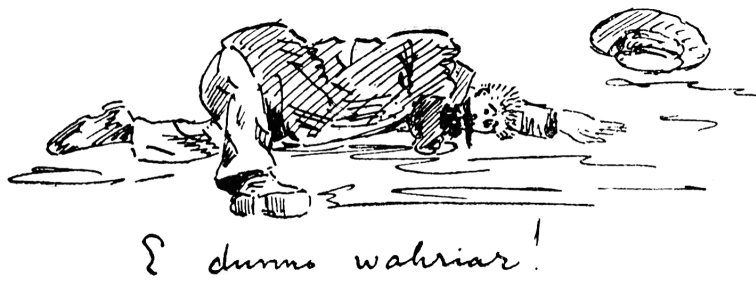
Charles Davies Sherborn. Self caricature after he had finished the last entry to the *Index Animalium* (from Norman, 1944). The handwritten quip “I dunno wahriar” is a stunned and worn-out Sherborn saying “I dunno where I are”.

However, *bona fide* members of the “Indexer’s Club” as defined herein – the ones that spend many years making lists of large groups of things – are not born that way but have, through experience with making a first list, found a unique form of satisfaction in making order out of something. It may not be as much the result of the efforts as the actual work of making order that is addicting or satisfying. Sherborn was one of these who found immense satisfaction from making lists of things, despite the incredible time and effort it often took (Fig. 1).

**Figure 2.**
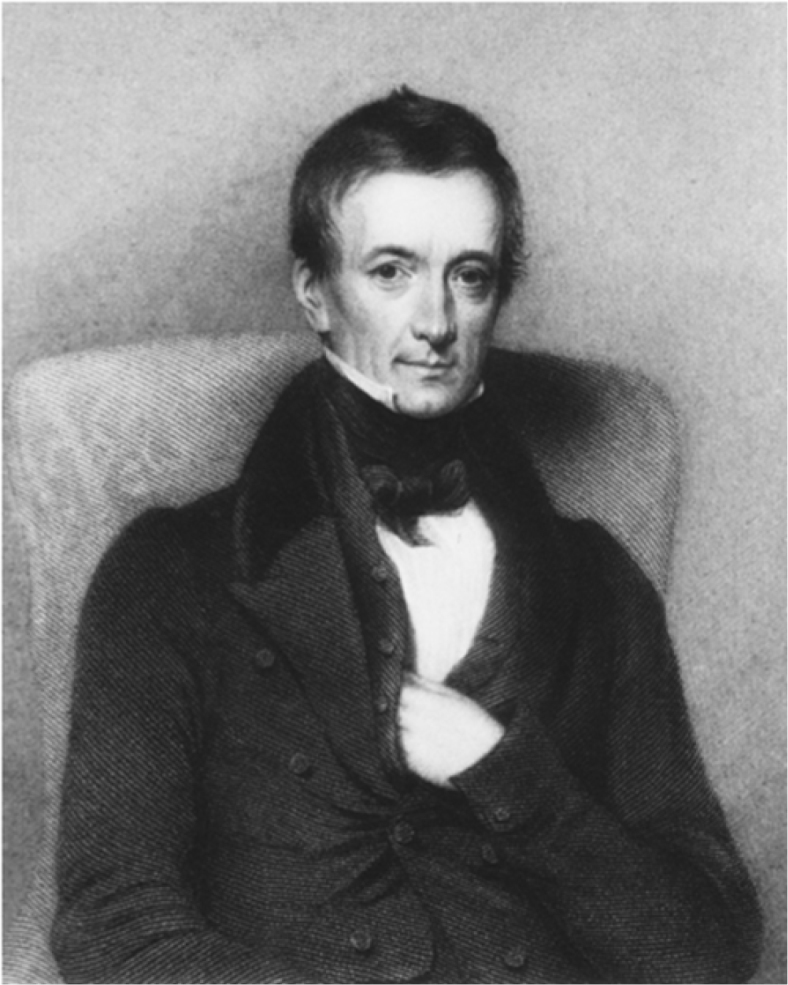
Peter Mark Roget (from Wikimedia Commons).

An indexer can naturally have a strong proclivity toward making lists but in some cases this obsession or addition may have come from an unhealthy or stressful background. Such was the case of one of the best-known list makers, the polymath Peter Mark Roget (1779–1869) (Fig. 2). Although his contemporaries thought he would be best known for his 2-volume, *Bridgewater treatise* on the physiology of plants and animals (Roget 1834), the work that would instead put his name into the vocabulary of millions upon millions and that would be referred to more than most dictionaries by undergraduate students worldwide was his *Thesaurus*, the work that is synonymous with synonyms. It is one of the most famous of all reference works, but few know of the life of its author or what drove him to list-making.

Like all indexers, Roget longed to put order into his world. Unfortunately, Roget’s world as a child had his father die prematurely, his beloved uncle commit suicide in his presence, and his maternal grandmother and mother each suffer from an unidentified mental illness. To escape this dark and dreary world, Roget comforted himself with words, and started making lists at the age of eight. One of his first lists was of his relatives and family and the dates of their deaths (Kendall 2008). From that morbid beginning, he went on to initially make lists of the things he found around him such as plants, animals, cloud types, and minerals, all with their Latin equivalents (Latin being a favorite subject of his, even at a very early age). He found solace in list-making and he didn’t stop. Throughout his life, he kept files of all the words and their synonyms that he could find. Incredibly, it wasn’t until he was 73 years of age before the first edition of *Roget’s Thesaurus of the English language* appeared on bookshelves (Roget 1852). His indexing was thus a life-long but immensely personal obsession.

But to stereotype an indexer as being like Roget can be dangerously wrong. Not all come from the extremely depressing and stressful background of Roget. Yes, Sherborn could be easily defined as a “workaholic”, involved often in a number of concurrent projects. Norman (1944) gave examples of Sherborn at various times during the production of his *Index Animalium* also working on other indexes such as the one of all the genera and species in Linnaeus’s *Systema naturae* (Sherborn 1899). But he also did bibliographies and associated dating research, biographies and obituaries, synthesized all the known natural history collections into a single resource (Sherborn 1940), compiled his own family’s genealogy [progress on work and enquiries for further material noted in Sherborn (1898) and final publication in Sherborn (1901)], and various and sundry other lists, all while keeping up with his hobby of collecting Byzantine bronze coins. Yet, despite his constantly keeping himself busy with projects, and in striking contrast to the dreary surroundings of Roget’s life, Sherborn’s life was much more on the “normal” side.

## A brief vignette into the formative years of Charles Davies Sherborn

Charles Davies Sherborn, was born on 30 June 1861 in Gunter Grove, Chelsea (near central London but considered a rather rural area at the time with large open fields), and was baptized at St. Luke’s Church, well known at the time as having been the venue for the marriage of Charles Dickens 25 years earlier. His father was Charles William Sherborn (1831–1912) [Charles Davies Sherborn always signed himself as “C. Davies Sherborn” possibly to disambiguate himself from his father, also a Charles], an etcher and engraver, especially known for his book plates, and his mother was Hannah Sherborn (née Simpson) (1829–1922). Charles was the eldest of five children (one having died in infancy).

Sherborn wrote a biography of his father (Sherborn 1912) and his description of him, also quoted in Norman (1944), echoes many of the same qualities that were said to have been of the younger Sherborn himself:

“My father was a robust person, about five feet nine inches high, easy-tempered and easy going, though intolerant of bores and politics, and strongly Protestant in his religious convictions. He went about little in Society, disliking formalities, and rarely entertained anyone at his own home.” (Norman 1944: 12).

In the only place he described himself, the genealogy of his family, Sherborn simply stated:

“Educated by Miss Elizabeth Rye and at St. Mark’s College School, Chelsea; was in business from 1876–84, when he went to Switzerland and Germany, afterwards devoting himself to the bibliography of the zoological and geological sciences” (Sherborn 1901: 142).

His early education was unremarkable but, after examinations, he did obtain from the South Kensington Museum (now the Victoria and Albert Museum) a certificate in geology which afforded him a life ticket to the library of that Museum. Geology being his favorite subject, this ticket to the library was heaven-sent and undoubtedly opened the door for his unquenchable thirst for knowledge. Having used the ticket often helped influence his philosophy toward education in that he felt that students should not be given facts, but should instead learn where to be able to find them (Norman 1944: 18). This self-professed credo of the essential tool in learning being the use of finding aids may have helped lead him toward his passion for indexing, as eventually Sherborn made his own finding aids for others to help empower them and expedite the search process, especially for things related to zoology.

Financial misfortune of his father’s business forced Sherborn to abandon his education at the age of 14 and he soon found a job at the bookseller’s and stationer’s shop of Frederick William Stockley (1872–1948). Sherborn immersed himself in his work and soon became familiar with every aspect of the book trade. His duties included tending to the shop, cataloguing the stock, and traversing the streets of London collecting the day’s orders of books, the last duty of which allowed him to find good bargains for his own personal book collecting. It is without a doubt that both his life ticket to the Museum library and his 6-year experience with the bookseller trade were to be linchpins in his future expertise with bibliography, dating research, and indexing. Although he was brought up with the financial hardships of his father’s business, Sherborn himself was prudent with money throughout his life and after he passed away in 1944, probate records have his effects listed in the amount of £11619 (Anonymous 2010), which is equivalent to over US$500,000 in 2014 currency.

Stockley’s bookselling business eventually went into bankruptcy in 1901 but before this, in 1883, Sherborn had left his employment in the bookselling trade to take on a few other odd jobs. The following year, Sherborn became employed by then-retired geologist and paleontologist Thomas Rupert Jones (1819–1911), and Charles’s professional career had now been set on course. This association with Jones ultimately led to a visit to the new natural history museum at South Kensington and meeting the many scientists in the Geology Department. Jones had employed Sherborn to help illustrate and finish some monographic works on Foraminifera. It was not long after his initial work with forams that Sherborn realized a good bibliography and index were essential to better understanding and study of them. His work on the foram bibliography began around 1886 and was published a few years later (Sherborn 1888).

His acquaintances made at South Kensington led to Sherborn being employed by the British Museum (Natural History) around this same time. He was initially contracted in the Geology Department to mount specimens, but he quickly became involved in indexing and bibliographic work.

After his bibliography of the Foraminifera came his index to its genera and species (Sherborn 1893–1896). In discussing the foram bibliography and index to his biographer J.R. Norman, Sherborn gave a quote that encapsulated his life-long obsession:

“I suppose that I must have a card-index mind, because the preparation of my *Bibliography* and *Index* of that group (which my friends considered to be incredibly dull) gave me a lot of pleasure.” (Norman 1944: 51).

Thus, with that first index, Sherborn was bitten by the indexing bug and never looked back. He was addicted. His life’s path had been chosen.

With his “card-index mind” in full gear, working on the bibliography and concurrent assembling of the card-index for the Foraminifera gave Sherborn an idea. He felt that what could be done for the forams could also be done for all of zoology: an *Index Animalium* that would give a complete listing of every genus and species name, author, and accurate date of publication. Whether or not he understood the immensity of the task, this work would, in essence, captivate much of his time for the next 43 years.

## A selected list of members of the Indexer’s Club

During those next 43 years, and even a few years afterwards, Sherborn was involved in three types of indexing: bibliographies, ascertaining correct dates of publication (i.e., putting publications in proper chronological order), and nomenclators (lists of names). Many of his predecessors in this Indexer’s Club who were involved in various types of indexing may well have been potential inspirations for him. Others in this Club may well have in turn been inspired by Sherborn in their own work.

Bibliographies make order of writings that otherwise would be scattered citations and it is one of the first forms of indexing. The Greek librarian Kallimachos (310–240 BC) “invented” the library catalog and was the first bibliographer (Blum 1991); and he is most famous for having made a bibliography of all the holdings of the Alexandrian Library. This listing was undoubtedly useful for users of the library, but Kallimachos unfortunately did not make a back-up copy and, when the Alexandrian Library was destroyed some 200 years after his death, the bibliography by Kallimachos was also destroyed, thus we will never know all that was in that library. Despite that unfortunate loss, the discipline of making bibliographies continues to this day and derives from the work of this man.

**Figure 3.**
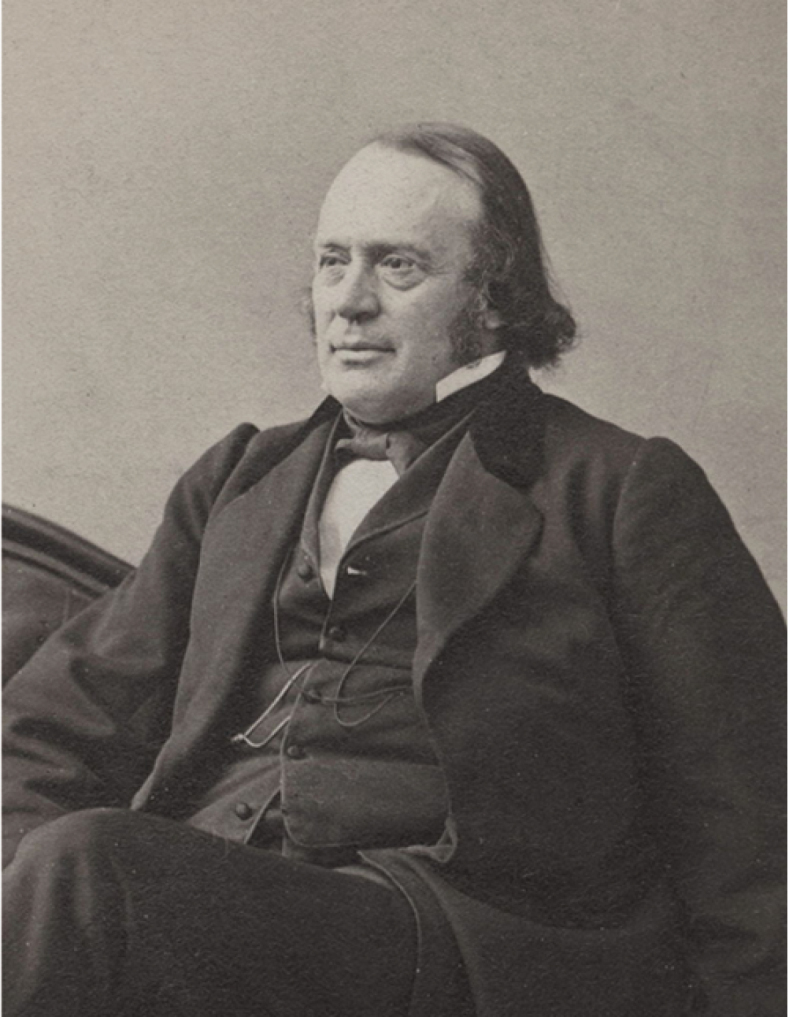
Louis Agassiz (from Wikimedia Commons).

Louis Agassiz (1807–1873) (Fig. 3), a geologist and paleontologist by profession, became well known for two large series of works. One was his 4-volume bibliography, *Bibliographica zoologiae et geologiae* in which he was assisted by H.E. Strickland and W. Jardine (Agassiz and Strickland 1848, 1850, 1852; Agassiz et al. 1854). The other was his *Nomenclator zoologicus*, a list of animal generic names in a series of 12 fascicles published from 1842 to 1846; a summary volume of all the generic names was published in 1846 as *Nomenclatoris zoologici index universalis* (Agassiz 1846).

**Figure 4.**
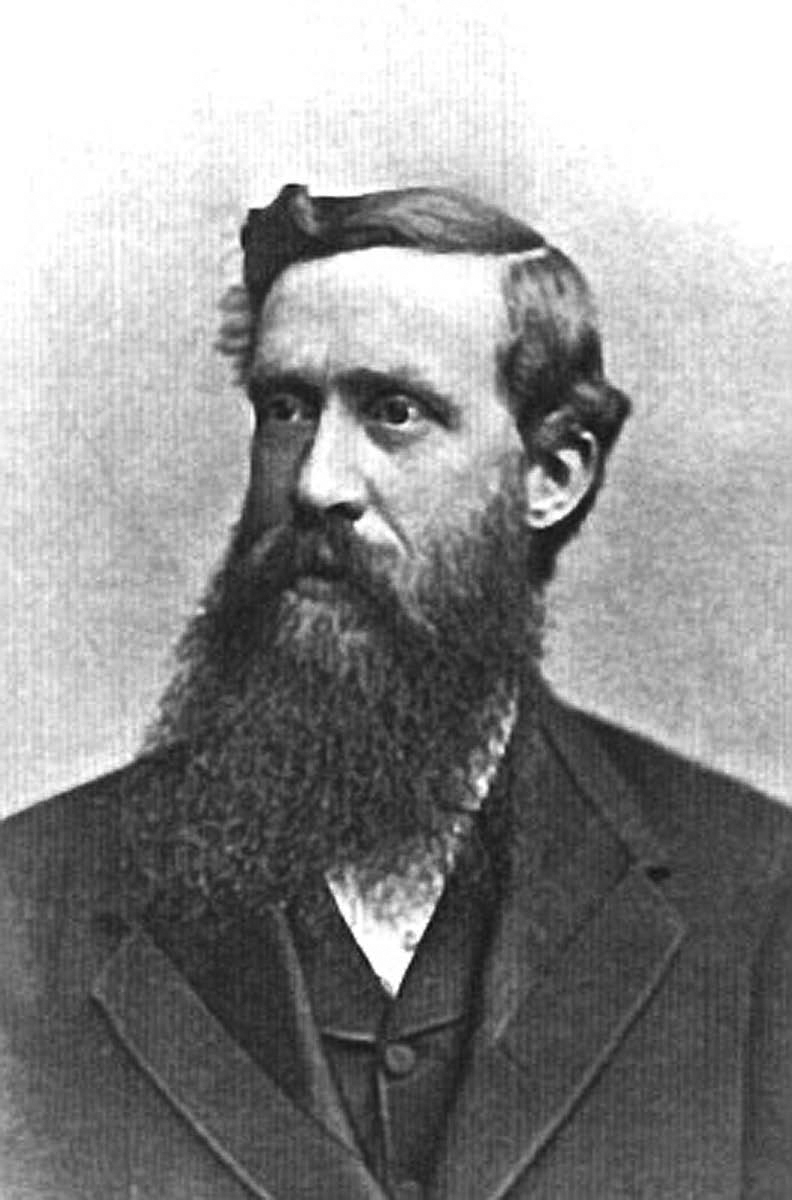
Elliott Coues (from Wikimedia Commons).

Another bibliographer who had conducted his research prior to Sherborn’s working on his *Index* was ornithologist Elliott Coues (surname pronounced “cows”, not “coos”) (1842–1899) (Fig. 4). Coues worked at the Smithsonian and produced a four-part bibliography of ornithology (Coues 1878, 1879, 1880a, 1880b). His bibliographic work was excellent and showed the labor necessary to provide users with an ordered set of articles. However, it was a quote by him that I feel is worthy of repeating here. I quoted only a portion of it in my own bibliography of Diptera books (Evenhuis 1997) but the full quote should have been reproduced there as it typifies what can easily happen to bibliographers like Sherborn, myself, and possibly others once we start making bibliographies:

“... bibliography is a necessary nuisance and horrible drudgery that no mere drudge could perform. It takes sort of an inspired idiot to be a good bibliographer and his inspiration is as dangerous a gift as the appetite of the gambler or dipsomaniac – it grows with what it feeds upon, and finally possesses its victim like any other invincible vice.” (Coues 1897: 39).

Coues was indeed addicted to bibliography and his fervent devotion to his work showed him to also do other types of indexing, such as also producing various checklists of North American birds. Some of these checklists were simple lists of common names and scientific names, but others came with classical language etymologies and sometimes even delved into proper orthoepy [correct pronunciation] (Coues 1882). Although it is a much-debated specialty, we need more works that research proper orthoepy in biology, Jaeger (1960) being one of the last major ones in that discipline.

**Figure 5.**
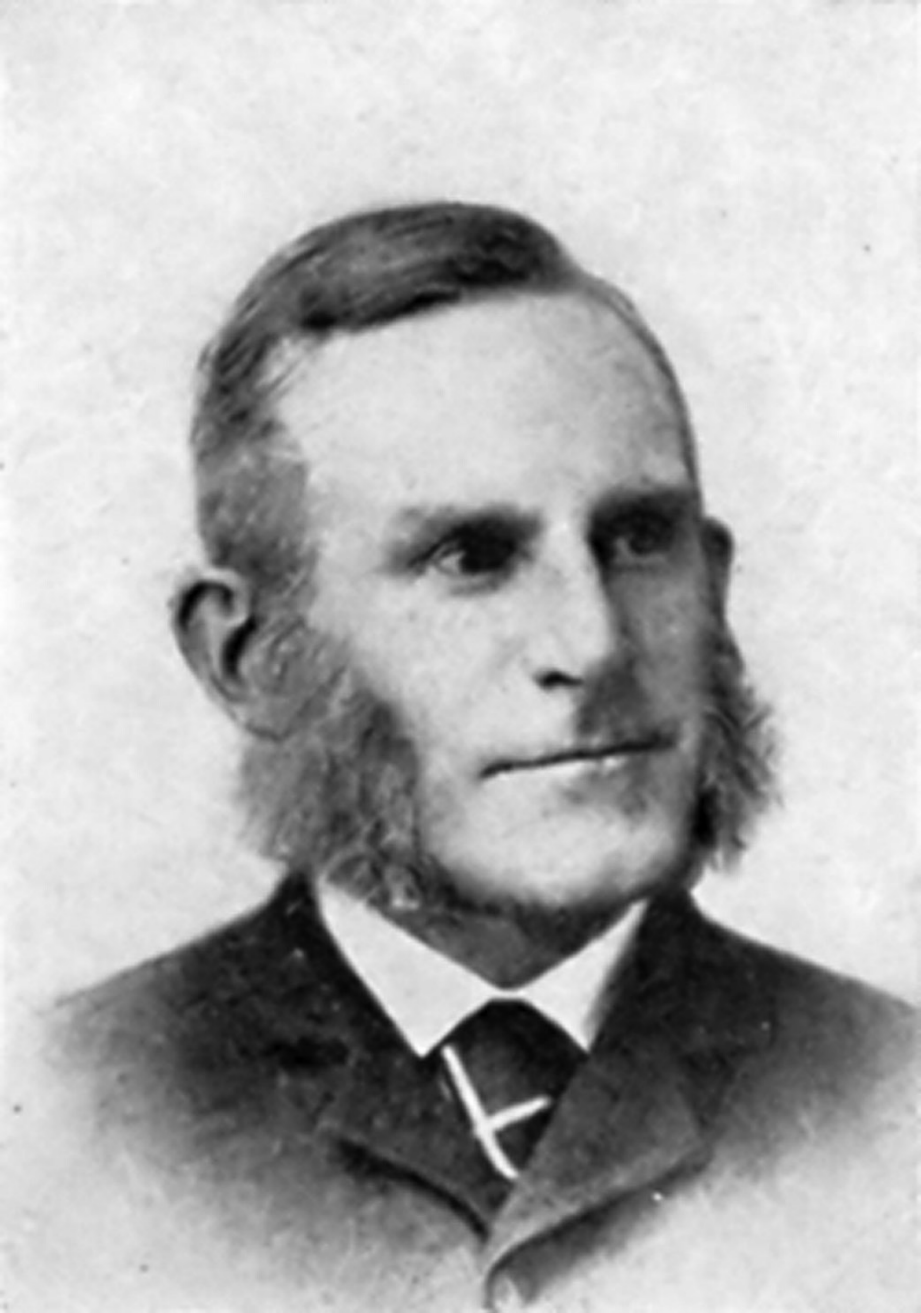
Samuel Hubbard Scudder (from Wikimedia Commons).

Another predecessor of Sherborn who compiled both bibliographies and nomenclators was Samuel Hubbard Scudder (1837–1911) (Fig. 5). Scudder was primarily an entomologist and, like Agassiz, was interested in paleontology. While he was assistant librarian in charge of the catalogue at Harvard College [he resigned this post on 1 December 1882 to become the first editor for some new-fangled journal called *Science*], Scudder compiled a serialized bibliography. However, he restricted it to the literature of fossil insects (Scudder 1880–1882). A later revised and annotated edition was published in Scudder (1890). But before he had even finished his first iteration of that bibliography in 1882, he had decided to assemble the names from the previous genus-group name nomenclators of Agassiz (e.g., 1846) and Marschall (1873) and bring the list up-to-date. By employing a team of colleagues worldwide who gave him lists of names in their discipline, he was able to provide the most complete list of genus-group names in zoology at that time in two parts (Scudder 1882, 1884). Scudder had indicated in his prefatory narrative that there would be periodic updates of this list but they never appeared. This absence of updates strained the patience of Scudder’s colleagues at the British Museum (Natural History). And since many of the curatorial staff there were already compiling names of animals each year for the *Zoological Record* and had these names at hand, C.O. Waterhouse, with the assistance of David Sharp, eventually provided an update to Scudder’s nomenclator to bring the generic names up to the year 1900 (Waterhouse 1902).

**Figure 6.**
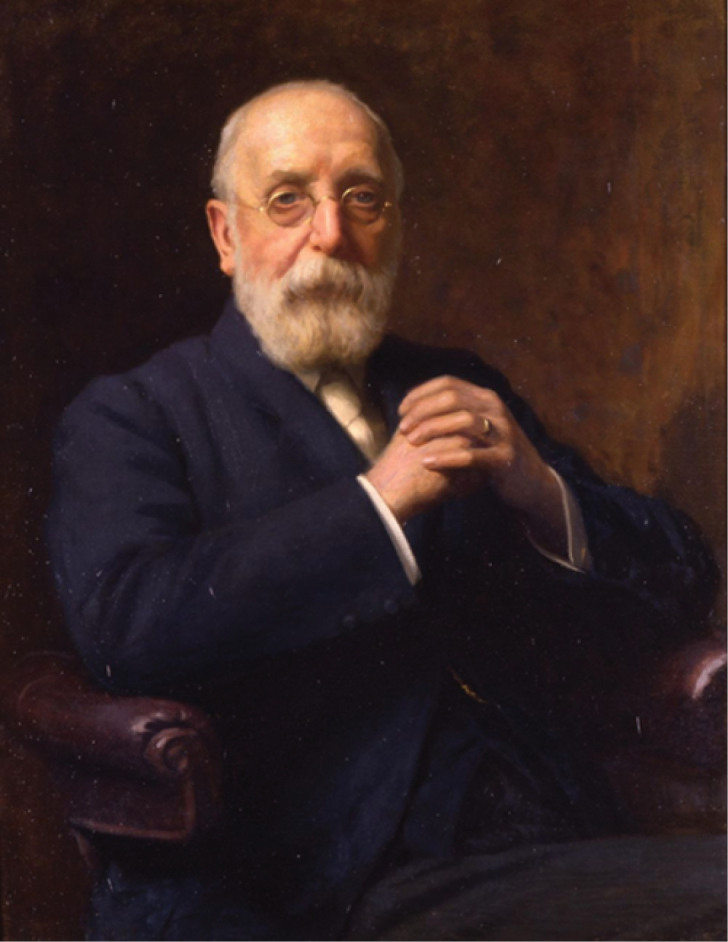
Benjamin Daydon Jackson (courtesy of the Zoological Society of London).

C.O. Waterhouse’s (1902) nomenclator and a list of bird names a few years earlier by another Waterhouse (Waterhouse 1889) were no doubt inspirations to Sherborn and probably assisted him in his production of the *Index Animalium*. But the single person who without a doubt had the most influence on Sherborn’s idea for an *Index Animalium* was Benjamin Daydon Jackson (1846–1927) (Fig. 6), who was busy working on the first nomenclator of plants, the *Index Kewensis* (Jackson 1895). Jackson was already immersed in his index when Sherborn had begun his contract work at the British Museum. Jackson’s list was not just of generic names but also of species names. It was the first list to bring together all the names known of plants at that time. If the plants could be done, why not the animals?

## Work on the *Index Animalium*

Sherborn began his work organizing his *Index* in the late 1880s and in May 1890 sent a letter to *Nature* (Sherborn 1890) and a similar one to *Le Feuille des Jeunes Naturalistes* outlining his proposed project and requesting advice and feedback from readers. After receiving responses from colleagues throughout Europe, Sherborn began work in earnest on 1 July 1890. Yearly progress reports were given to the British Association for the Advancement of Science in the early going and after six year’s of work, some 130,000 slips with names had been catalogued by Sherborn (Sherborn 1896a).

The methodology employed by Sherborn for his *Index* is exemplary of anyone who wishes to produce an accurate and complete list or database of names. He avoided perpetuating potential errors by others by not working from previous lists. Instead he examined each original publication, scanning each page and writing the binomials he found on two slips of paper: one for the alphabetical index by species; the other for the index by genus. Sherborn’s methodology was painstakingly tedious but was the only way to ensure all names in a particular publication would be captured in his *Index*. Despite the rigor that went into this form of data entry, Sherborn’s work is not without its errors and omissions. Poche (1939) gave a list of over 2,000 names that were missed by Sherborn, mostly malacological and subspecific or varietal names, but most were names already rejected by ICZN action in 1912, so are of little value to malacology. Welter-Schultes et al. (2016) gives further details on other errors in Sherborn’s *Index*. Nevertheless, the incredibly small error rate for some 440,000 hand-written names from examination of nearly 28,000 books and articles over 43 years of work is indeed an astonishing feat that will probably never be duplicated.

## Bibliographies

Both indexing and bibliographies were an important part of the work that occupied Sherborn for almost half of his life. His own words exemplify their need:

“The systematist requires certain tools for his work, of which not the least important are good bibliographies and indexes.” (Norman 1944: 49).

We have already mentioned Sherborn’s first bibliography on Foraminifera, but he obviously did not stop with that. Between 1888 and 1895 a “Bibliography of Malaya” appeared in serial form in the *Journal of the Straits Branch of the Royal Asiatic Society*. Other subsequent bibliographies included a list of natural science reference works (Sherborn 1894); a catalogue of the Linnaean Society Library (Sherborn 1896c); the writings of Gilbert White (Sherborn 1900); the conchological writings of Thomas Brown (Sherborn 1905); a bibliography of scientific literature relating to Egypt (Sherborn 1910, 1915); and a summary of natural history dating sources (Sherborn et al. 1936).

## Dating works

Soon after beginning his work on compiling bibliographies, Sherborn realized the necessity for obtaining accurate dates of publication for the works he was listing. As Conrad (1853) stated in the prefatory sentences to his list of North American “Naiades”:

“To render strict justice to every author according to date of publication, is not only the duty of the naturalist, but a necessity of science.” (Conrad 1853: 243).

Sherborn’s first article on dating (Sherborn 1891) was published soon after beginning his compilation of the first *Index Animalium* and an article pleading for the need for proper metadata on each publication of books was published a few years later (Sherborn 1896b). These studies on accurate dating were not only critical in resolving dating problems of some early zoological works that had never had been put into context with other works of that same period in time, but, in order to ascertain priority among taxonomic names, accurate dates of their original proposals were required. A number of dating articles of other early works soon followed his original 1891 paper and culminated in giving accurate dates in his bibliography that accompanied the first *Index Animalium* (Sherborn 1902).

Publication of the first *Index* did not stop Sherborn’s work on dating since he needed to resolve further problems of dating for works that were to appear in his second *Index*. No fewer than 20 articles on dating or publication histories by Sherborn and various co-authors appeared between publication of the first *Index* and the publication of the last part of the second *Index*. As with his first *Index*, the bibliography of his second *Index* also was replete with proper dating.

Despite his *Index Animalium* being completed in 1933, Sherborn, being the consummate facilitator and indexer, did not stop being concerned with proper dates of publication and, with the assistance of bibliographer Francis James Griffin (1904–1990) and Kew Gardens librarian H.S. Marshall, published a synthesis of published sources that focused on bibliographical research and gave dates of publication for biological works. Their paper appeared as the first article in the first issue of the *Journal of the Society for the Bibliography of Natural History* (Sherborn et al. 1936), the society of which was founded by a group of fellow bibliographers who had an interest in seeing the results of bibliographic and dating research to be made public via a scientific journal. Not surprisingly, Sherborn was the Society’s first president, typifying the zeal he had for this subject and also typifying his desire to further facilitate such information to fellow bibliographers and taxonomists worldwide. The formation of this society could be said to have been the beginnings of a formalized group of individuals who were interested in the discipline that would later be called “bioinformatics”.

## Influence on and future of bioinformatics

Sherborn did indeed construct a solid foundation for the future with his seminal works on bibliography, dating, and indexing. His works were followed by many others, either providing indexes, bibliographies, or catalogues, on small groups of organisms such as by family or country, or larger, more comprehensive studies. By way of a few examples, I will list a few of some of the more major works that have been produced since Sherborn’s *Indexes* and inspired by his vision.

### Nomenclator Zoologicus

**Figure 7.**
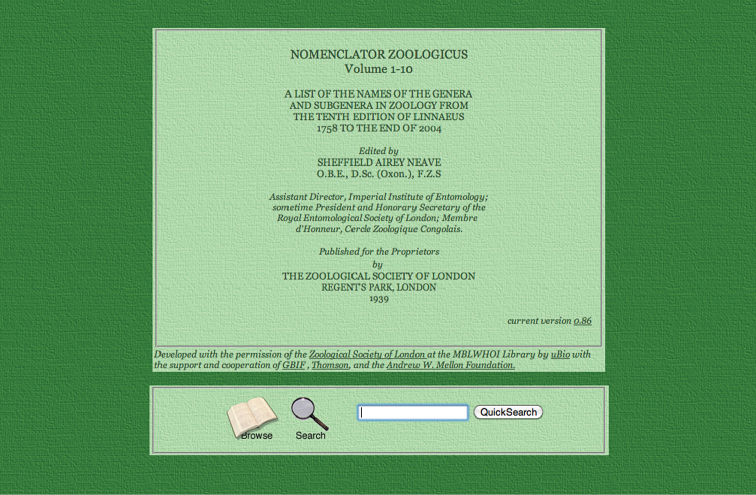
Screenshot of *Nomenclator Zoologicus* online.

Sheffield Airey Neave (1879–1961), while working at the Imperial Bureau of Entomology, recognized the need for up-to-date information on all generic names in zoology and envisioned a nomenclator to index all of them. With initial funding from the Zoological Society of London, the original edition of his *Nomenclator Zoologicus* in four volumes (Neave 1939–1940) was published. Five supplement volumes appeared until 1994, but the absence of further supplements is not as devastating as originally thought by some. The hard-copy volumes, which used to be a major reference for any taxonomist wishing to describe a new genus and needing to check whether or not that name was used before, have all been digitized. With funding to a few dedicated staff from Thompson-Reuters, GBIF, and the Mellon Foundation, and partnering with the Zoological Society of London, over 340,000 genus-group names in the *Nomenclator Zoologicus* are now available online: http://uio.mbl.edu/NomenclatorZoologicus/ (Fig. 7).

### Catalog of Fishes

In the 1980s, William N. “Bill” Eschmeyer (1939– ), ichthyologist at the California Academy of Sciences, decided to organize all the taxonomic information on ichthyology. With initial partial funding from the National Science Foundation (and later technical support from the California Academy of Sciences), he began a task some thought impossible: to catalogue all the genera and species of fishes worldwide. Undaunted, his first volume on genus-group names appeared in 1990 (Eschmeyer and Bailey 1990) and eight years later his dream was fulfilled in the publication of his three-volume catalog of all genus- and species-group names of fishes and an associated bibliography (Eschmeyer 1998). He had followed the methodology of Sherborn in not only examining all the original literature, but made painstaking efforts to obtain accurate dates of publication for these works. But Bill was aware that hard copies of his work would not meet the needs of everyone, and the internet showed him the potential to reach workers who did not have the resources to purchase his volumes. He did not miss the opportunity and made available to the public all of the information he had compiled into a database over the years via a simple and user-friendly web interface [http://researcharchive.calacademy.org/research/Ichthyology/catalog/fishcatmain.asp]. The database is continually updated and is the best one-stop shopping for fish names and literature resources anywhere.

### *Systema Dipterorum*

In 1984 at the XVII International Congress of Entomology in Hamburg, a group of dipterists working at the U.S. Department of Agriculture’s Systematic Entomology Laboratory in Washington, DC proposed a plan to database all the names of two-winged flies (Diptera), which it turns out comprise a fairly large percentage of all animal names (15%). A great deal of interest was spurred from that presentation but, aside from a grant from GBIF in 2003, support through 4D4Life in 2009, the CoL Rotating Fund in 2010, and small yearly grants from the Schlinger Foundation during the last few years, meager funding over the years supports the time and staffing necessary to maintain and complete the project and funding ceased altogether in the last two years. This has not deterred F. Christian Thompson (1944– ) from seeing this vision to fruition. The ensuing 25-some odd years since the announcement in 1984 saw Chris working diligently in the evenings in his home office to continually enter, update, and verify data in the *Systema Dipterorum*
(SD). Thompson and Pape (2016) give further details on the history and status of SD, and as of October 2013, 4,653 family-group names (most catalogued in Sabrosky 1999), 23,437 genus-group names, and 198,258 species-group names in 32,900 published works have been entered into SD and are available for searching via a robust web interface at [www.diptera.org] (see also Pape and Thompson 2010). Because of the high standards put onto the methodology of data acquisition, entering, and vetting, and because data vetting follows the rigorous example set by Sherborn in his *Index Animalium* of examining every original paper, the SD is today one of the most accurate and complete databases of any megadiverse group of animals.

### *Index Animalium* Online

**Figure 8.**
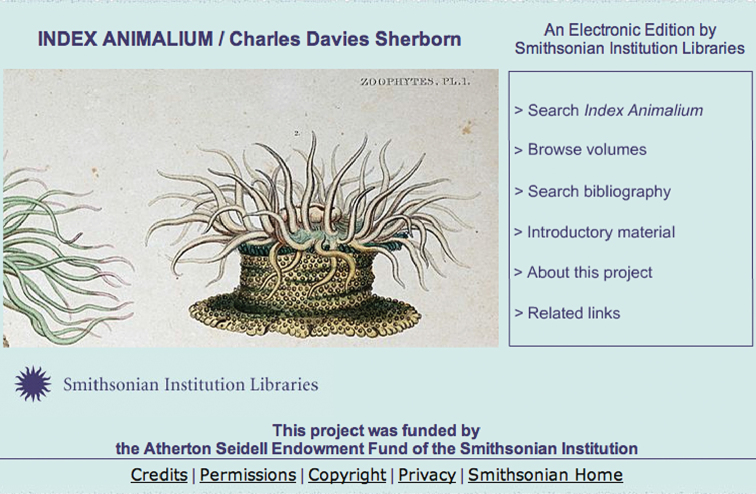
Screenshot of *Index Animalium* online.

With exemplary foresight, in 2004 the Atherton Seidell Endowment Fund at the Smithsonian Institution brought the work of Sherborn into the 21^st^ century by recognizing the importance of Sherborn’s *Index Animalium* and making it available to as wide array of users as possible. It funded both the digitization of both editions, data parsing and re-keying, and design and implementation of the user interface on the web [http://www.sil.si.edu/digitalcollections/indexanimalium/TaxonomicNames/] (Fig. 8) allowing searching of every name that occurs in every part that was published from 1902 to 1933. Pilsk et al. (2016) give details on the labor that went into the digitization and parsing data on each page, with the goal of achieving a 99.995% accuracy rate in converting the OCR text.

### The Future

It is the internet, and whatever iterations it evolves into, that is and will be the medium for making available the information we need on all aspects of cataloguing, nomenclature, bibliography, and dating. The final few papers in this volume (e.g., Penev et al. 2016; Page 2016; Pyle 2016) deal with how the names of animals and the information and metadata associated with them can be standardized and implemented for universal access. Although there is much yet to be done, we are making significant progress in serving up the information on biological names for future generations through immediately accessible electronic media.

Sherborn could never have dreamed that his small slips of paper with names hand-written on them would be replaced by 1s and 0s in binary form so that they could be transmitted electronically through an electronic medium that would have a viewing screen on everyone’s desk or handheld device. But he can be comforted that his tireless work of 43 years in producing his *Index Animalium* has had a profound influence on what we do today to facilitate the research of others in studying biological taxa and the names associated with them.
